# Survival and time-to-transplantation of peritoneal dialysis versus hemodialysis for end-stage renal disease patients: competing-risks regression model in a single Italian center experience

**DOI:** 10.1007/s40620-016-0366-6

**Published:** 2016-11-29

**Authors:** Marta Rigoni, Emanuele Torri, Giandomenico Nollo, Diana Zarantonello, Alessandro Laudon, Laura Sottini, Giovanni Maria Guarrera, Giuliano Brunori

**Affiliations:** 1 Innovazione e Ricerca Clinica in Sanità – IRCS, Fondazione Bruno Kessler, Via Sommarive, 18, 38123 Trento, Italy; 2 Dipartimento Salute e Solidarietà Sociale, Provincia Autonoma di Trento, Trento, Italy; 3 Biotech, Dipartimento di Ingegneria Industriale, Università di Trento, Trento, Italy; 4 U.O. Nefrologia, APSS Trento, Trento, Italy; 5 Direzione Sanitaria APSS Trento, Trento, Italy

**Keywords:** Dialysis survival, Hemodialysis, Advanced chronic kidney disease, Kidney transplantation, Peritoneal dialysis, Competing-risks model

## Abstract

**Aims:**

Despite several studies reporting similar outcomes for peritoneal dialysis (PD) and hemodialysis (HD), the former is underused worldwide, with a PD prevalence of 15% in Italy. In 2008, the Unit of Nephrology and Dialysis of the Healthcare Trust of the Autonomous Province of Trento implemented a successful PD program which has increased the proportion of PD incident patients from 7 to 47%. We aimed to assess the effect of this extensive use of PD by comparing HD and PD in terms of survival and time-to-transplantation.

**Methods:**

A total of 334 HD and 153 PD incident patients were enrolled between January 2008 and December 2014. After screening for exclusion criteria and propensity score matching, 279 HD and 132 PD patients were analyzed. Survival and time-to-transplantation were assessed by competing-risks regression models, using death and transplantation as primary and competing events.

**Results:**

Crude and adjusted regression models for survival revealed the absence of significant differences between HD and PD cumulative incidence functions (subhazard ratio: 1.09, p = 0.62 and 1.34, p = 0.10, respectively). Differently, crude and adjusted regression models for transplantation revealed a lower time-to-transplantation for PD versus HD patients (subhazard ratio: 2.34, p < 0.01, and 2.57, p < 0.01, respectively). The waiting time for placement in the transplant waiting list was longer in HD than PD patients (330 vs. 224 days, p < 0.01).

**Conclusions:**

The extensive use of PD did not lead to any statistically significant difference in mortality. Furthermore, PD was associated with lower time to transplantation. PD may be a viable option for large-scale dialytic treatment in the advanced chronic kidney disease population.

## Introduction

Hemodialysis (HD) and peritoneal dialysis (PD) are the two common forms of dialysis therapy for end-stage renal disease (ESRD). Although PD is a well-established treatment modality for advanced chronic kidney disease offering several potential benefits, it is underused in Western countries [1]. In 2008, only 11% of the dialysis population was treated with PD worldwide [2]. PD prevalence varies significantly between regions. The proportion of dialysis patients on PD reaches the 79% in Hong Kong [2]. Conversely, PD initiation in the United States has traditionally been low, never exceeding 15 to 16% of incident or prevalent maintenance dialysis patients [1]. In Italy, recent data from the Italian Study Group of Peritoneal Dialysis have reported an incidence of PD modality of approximately 20% with a prevalence of 15% [3]. The underuse of PD does not seem justified in terms of safety and effectiveness; it could be explained, instead, by a complex set of clinical, organizational, economic as well as patient-related factors favoring HD use [4]. Indeed, data derived from both national registries and large observational studies agree that the survival rate of patients treated with both modalities is at least comparable [5, 6].

A randomized controlled clinical trial on dialysis modality was attempted in the Netherlands, but only 38 of 773 eligible subjects provided consent to be randomized to receive the different treatment options [7]. Currently available survival data are virtually all observational and thus affected by confounding factors and limitations [8]. Indisputable evidence for beneficial effects on survival can be obtained only from randomized clinical trials, but it is unrealistic to expect such an initiative in the near future [9]. Therefore, the acquisition of reliable and comprehensive observational data is of the utmost importance to reliably examine the clinical consequences of radical modifications in dialysis therapy [9]. Over the last 10–15 years, as overall survival of dialysis patients has steadily improved and statistical methods to analyze observational data have evolved, a pattern of virtual equivalence in survival between patients on HD versus PD has emerged [8]. Previous studies showed that age ≥65 years, frailty, cardiovascular disease, and diabetes mellitus could worsen survival outcomes in PD compared to HD patients [10–12]. However, focusing on transplantation outcomes, PD patients could benefited from a higher rate of kidney transplantation and shorter time-to-transplantation with respect to their HD counterparts [13].

Further research is needed to improve PD uptake, and to support appropriate and patient-centered decision-making in the real care context to improve patient survival and well-being. The use of advanced statistical analysis techniques may affect the quality of studies and has the potential to highlight the most effective strategies for improvement.

Since 2008, a concerted effort has been made in the healthcare services of the Autonomous Province of Trento to expand the use of PD through the implementation of a capacity building program [14]. Following the introduction of this strategy, the incidence of PD patients progressively increased to 47% in 2013. Significantly, the prevalence of PD patients grew to 20%, which is higher than the Italian healthcare system average. Based on these results, the present study was designed to analyze the outcomes related to the increased use of PD over the 7-year program, and compare PD and HD in terms of patient mortality and time-to-transplantation.

To overcome limits due to the retrospective design of the study, a propensity score model was implemented to assure matching of the two treatment groups. Furthermore, survival data in ESRD patients were analyzed by competing-risks regression [15], since in these patients death and transplant act as competing events (i.e. the occurrence of one event hinders or modifies the occurrence of the other).

## Methods

### Treatment center

In 2008 the new clinical leadership of the Nephrology and Dialysis Unit and the implementation of evidence-based policies led to the initiation of a large-scale PD program. The program involved physician training in the use of PD, systematic pre-dialysis information and education [14], and clinical path standardization across the province. The management change was supported by promoting teamwork, collaboration, and performance feedback.

### Data collection and patients

This study was an observational, retrospective cohort study. Data were collected from 487 patients treated at the Unit of Nephrology of the Santa Chiara Hospital in Trento from January 1, 2008, to December 31, 2014. Patients were observed until September 30, 2015, granting a minimum follow-up period of 9 months. Patients decided to undergo treatment on a voluntary basis, after a detailed informative discussion and clinical evaluation with physicians and nurses: 334 patients chose HD and 153 PD treatment. All patients gave informed consent for the collection and processing of data in anonymous form. Exclusion criteria were: previous transplantation, or death within 30 days from the start of the treatment (i.e. in the adjustment to therapy period). Patients who chose to change dialysis treatment modality during the study period were censored. To correct the analysis for baseline covariates that could potentially affect the choice of dialysis modality, a propensity score model was implemented [16]. The estimation of the propensity score was performed with a logistic regression model based on region of common support including gender, age-group, cardiovascular disease, diabetes, chronic obstructive pulmonary disease (COPD), chronic liver disease, cancer, and hypertension as included variables. The matching between HD and PD patients was done with the Kernel Matching method. Distribution of baseline covariates before matching was not uniform for diabetes mellitus, while after propensity score matching it was uniform for all covariates. Demographic characteristics, survival, and comorbidities data were collected at the start of the dialytic therapy and derived from the Provincial Register of Dialysis Patients.

### Statistical analyses

Survival analysis was performed using competing-risks regression models, according to the method of Fine and Gray [17]. The model is based on cumulative incidence functions and subdistribution hazards risk (SHR) functions. For the survival study, the event of interest was death and the competing event was transplantation. Crude and adjusted regression models were computed. Adjustment was performed for age-group (<65 vs. ≥65 years), cardiovascular disease, diabetes mellitus, and arterial hypertension.

In order to evaluate the efficiency of the transplant process (i.e. time to placement on the transplant waiting-list, and time-to-transplantation), competing-risks regression models were calculated, considering transplant as the event of interest and death as the competing event. Crude and adjusted by age-group regression models were calculated. In the subgroup of patients on the transplant waiting list (54 HD patients and 49 PD patients) differences in waiting time for placement in the list and in call time-to-transplantation after placement in the list were assessed by Mann–Whitney U test. Data were expressed as median and interquartile range (IQR) for not normally distributed data, and number and percentage for categorical data. Categorical data were compared by the chi-squared test and continuous data by the Mann–Whitney U test.

A p-value less than 0.05 indicated statistical significance. All analyses were performed with Stata statistical software, version 13.0 (StataCorp, Texas 77845 USA).

## Results

### Patient characteristics and clinical data

Following screening for exclusion criteria and propensity score matching, 279 HD patients and 132 PD patients were analyzed. The characteristics of the two groups of patients after the propensity score matching are shown in Table 1. The two groups were uniform for each of the considered features. The glomerular filtration rate at the start of dialysis was between 4.5 and 6 ml/min (Chronic Kidney Disease Epidemiology Collaboration formula). The Kt/V ratio (urea clearance multiplied by treatment time/urea distribution volume) was maintained in the range of the Kidney Disease Outcomes Quality Initiative (KDOQI) guidelines (>1.2 per session in HD, 2.0 per week in PD). Regarding HD, 204 of 279 HD patients (73%) were treated by fistulas or grafts, and 75 (27%) by tunneled central venous catheter. Regarding PD, 86 of 132 PD patients (65%) adopted automated peritoneal dialysis, while 46 (35%) practiced continuous ambulatory peritoneal dialysis. More than 90% of the patients were put on dialysis after a period of pre-dialysis education and care at the hospital outpatient clinic.

**Table 1:** Characteristics of the study population after propensity score matching

	HD, n (%)	PD, n (%)	p
Total patients	279	132	
Males	194 (70)	90 (68)	0.78
Females	85 (30)	42 (32)	0.78
Cardiovascular disease	126 (45)	56 (42)	0.60
Diabetes mellitus	94 (34)	33 (25)	0.08
COPD	44 (16)	20 (15)	0.87
Chronic liver disease	29 (10)	15 (11)	0.77
Cancer	41 (15)	19 (14)	0.94
Arterial hypertension	234 (84)	105 (80)	0.28
Age at start of dialysis <65 years	107 (38)	56 (42)	0.43
Age at start of dialysis ≥65 years	172 (62)	76 (58)	0.43
Median age at start of dialysis, years [IQR]	69 [59–78]	69 [54–76]	0.20

*COPD* chronic obstructive pulmonary disease, *HD* hemodialysis, *IQR* interquartile range, *PD* peritoneal dialysis

### Survival analysis—death as event of interest

**Fig. 1:**
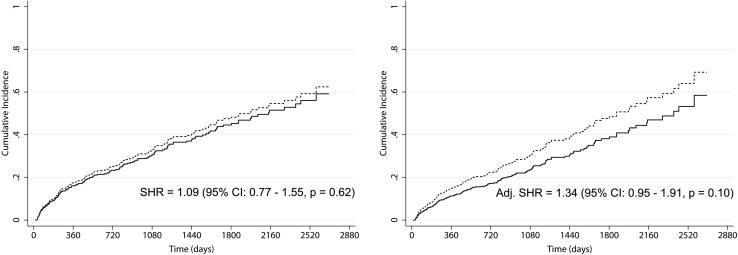
Cumulative incidence function of hemodialysis (HD, *continuous line*) and peritoneal dialysis (PD, *dashed line*) patients from 2008 to 2014, provided by crude (*left*) and adjusted (*right*) competing-risks regression models. In the model the event of interest was death and the competing event was transplantation. Adjustment was performed for age-group, cardiovascular disease, diabetes mellitus, and arterial hypertension

Death occurred in 102 (37%) HD patients versus 46 (35%) PD patients. As shown in Fig. 1, the cumulative incidence of death was slightly but not significantly higher in PD patients than in HD patients (SHR = 1.09, p = 0.62). The risk of death in PD patients did not change significantly (SHR = 1.34, p = 0.10) after adjustment for age-group, cardiovascular disease, diabetes mellitus, and arterial hypertension. Adjusted regression model values with covariate contributions are reported in Table 2. Aging (≥65 years) and cardiovascular disease increased the SHR of death, while arterial hypertension decreased it. Diabetes mellitus had no statistically significant effect. However, the confounders did not significantly modify the risk of death for PD patients in comparison to HD patients.

**Table 2:** Subdistribution hazard ratios of PD patients compared to HD patients according to the multivariate competing-risks regression model

Adjusted competing-risks regression—event of interest death		
	SHR (95% CI)	p
PD compared to HD	1.34 (0.95–1.91)	0.10
Age ≥65 years	4.22 (2.59–6.88)	<0.01
Cardiovascular disease	2.08 (1.42–3.04)	<0.01
Diabetes mellitus	1.17 (0.82–1.65)	0.39
Arterial hypertension	0.46 (0.30–0.71)	<0.01

*CI* confidence interval, *HD* hemodialysis, *PD* peritoneal dialysis, *SHR* subdistribution hazard ratio

### Survival analysis—transplantation as event of interest

**Fig. 2:**
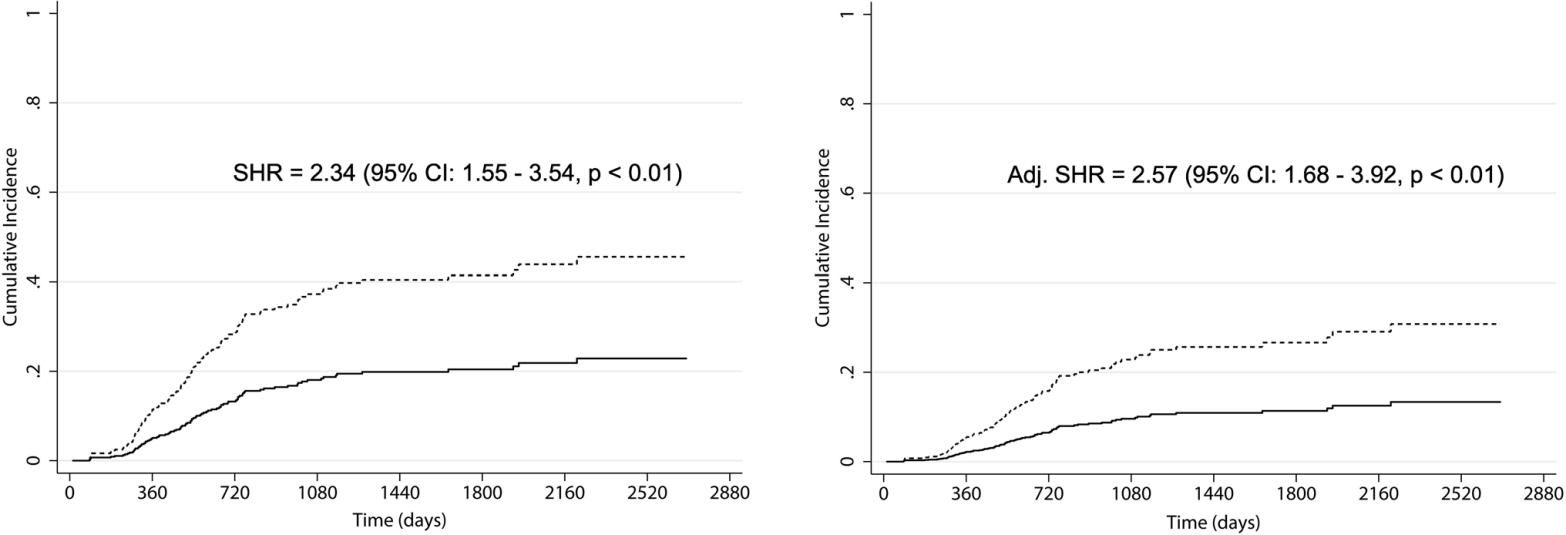
Cumulative incidence function of hemodialysis (HD, *continuous line*) and peritoneal dialysis (PD, *dashed line*) patients from 2008 to 2014, provided by crude (*left*) and adjusted (*right*) competing-risks regression models. In the model the event of interest was transplantation and the competing event was death. Adjustment was performed for age-group

Transplantation occurred in 42 (32%) PD patients compared to 47 (17%) HD patients. As shown in Fig. 2, the cumulative incidence of transplant was significantly higher in PD patients than HD patients (SHR = 2.34, p < 0.01). After adjustment for age-group, the SHR increased to 2.57 (Table 3). At multivariate analysis, age ≥65 years significantly decreased the likelihood of receiving a transplant (Table 3).

**Table 3:** Subdistribution hazard ratios of PD patients compared to HD patients according to the multivariate competing-risks regression model

Adjusted competing-risks regression—event of interest transplantation		
	SHR (95% CI)	p
PD compared to HD	2.57 (1.68–3.92)	<0.01
Age ≥65 years	0.06 (0.03–0.12)	<0.01

*CI* confidence interval, *HD* hemodialysis, *PD* peritoneal dialysis, *SHR* subdistribution hazard ratio

### Time-to-transplantation

A greater proportion of PD patients (49/132, 37%) were on the transplant waiting list than HD patients (54/279, 19%). The median time for placement on the transplant waiting list was significantly longer (p < 0.01) in HD [330 days (IQR 222–663)] than PD patients [224 days (IQR 178–363)]. The median time to receive the call for transplantation after placement in the waiting list was slightly but not significantly (p = 0.35) longer in HD [216 days (IQR 107–424)] than in PD patients [155 days (IQR 108–322)].

## Discussion

Broadening the evidence using comparative studies of dialysis outcomes and their impact on ESRD is essential to drive changes in care patterns and to help the nephrology community reflect critically on its practices. The present study aimed to evaluate the effects of an increased use of PD in the Autonomous Province of Trento in terms of survival and time to transplantation. This was performed within a framework of outcome assessment to improve the quality of care and quality of life of patients. In this study we analyzed data from 279 HD patients and 132 PD patients over a period of 7 years with a minimum follow-up of 9 months. Since the study was an observational retrospective cohort study, the power of the results was potentially limited.

It is well known that the comparison of two different treatments under real life conditions (i.e. outside the context of a randomized clinical trial) is affected by the ‘confounding by indication’ problem, which needs to be properly addressed. Therefore, we constructed a propensity score model [16] to match PD and HD patients. The score allowed us to analyze the baseline covariates that could potentially affect the choice of dialysis modality and thus to balance the risk of positive selection of the PD population. After propensity score matching we obtained two large patient groups, which were homogenous regarding clinical characteristics, modality and length of treatment.

The comparative study was performed using competing-risks regression models. Accounting for competing risk events permits the simultaneous analysis of outcomes and avoids overestimation of cumulative incidences [15]. In our study, the competing-risks model was applied twice: considering death as the event of interest and transplantation as the competing event, and vice versa. Survival analysis did not reveal any evidence of difference between PD and HD in terms of mortality. Consistently, crude and adjusted regression models for survival revealed no significant difference in terms of cumulative incidence functions between patient groups.

Although disputed by a recent analysis [18], the literature generally reports better survival for PD patients compared to HD patients during the first years of treatment [19]. Our study performed on two samples equivalent for frailty and complex disease characteristics did not confirm this finding. Indeed, PD patients showed a proportional and slightly higher incidence of death than HD patients during the whole treatment course, but without any statistically meaningful difference.

Aging (≥65 years) and cardiovascular disease increased the SHR of death. However, these confounders did not significantly modify the risk of death in PD patients with respect to HD patients. The issue of aging and cardiovascular disease in PD patients is still debated in the literature [20]. Some studies conducted with different methods reported a higher risk of death in PD patients than HD patients, which increased with aging and cardiovascular disease [10, 21, 22]. In contrast, Buemi et al. reported that in elderly and cardiac patients, PD was actually preferable, because in comparison with HD it reduced the hemodynamic stress experienced by the patient and the incidence of hypotension [23].

Although diabetes is recognized as a confounding variable able to affect survival in PD versus HD patients, our study did not result in an increased risk of death for ESRD patients. A recent systematic review analyzed mortality outcomes in diabetic patients who underwent HD or PD. The analysis of 25 observational studies led to the conclusion that the available evidence was inconsistent, because survival varied across study designs, follow-up periods, and patient subgroups [12].

Our results showed that hypertension decreased the risk of mortality in ESRD patients, but it did not affect the survival differences between the two dialytic treatments. The explanation for this finding is still controversial. Some reports have indicated a paradoxical association between hypertension and mortality in hemodialysis patients. According to this, a normal to low blood pressure seems associated with poor outcome, whereas high pressure potentially confers survival advantages, a phenomenon termed ‘reverse epidemiology’ [24].

Hence, confounders analysis in a real clinical context showed that major clinical complications did not change the cumulative incidence of death in the two patient groups in a meaningful way. Indeed, the two treatment modalities displayed a substantial equivalence, apart from a small non-significant negative trend in the PD group.

With respect to transplantation, the literature shows that PD commonly registers a higher rate of kidney transplantation [25]. According to data from the Italian national registers, this result seems to be due to the younger age of PD patients and the higher prevalence of first dialysis experience [26]. In our study, where the number of patients who could undergo PD was enlarged and patient age was comparable between the two groups, PD nonetheless had a reduced time-to-transplantation. Crude and adjusted regression models for transplantation revealed a significantly lower time-to-transplantation for PD compared to HD.

According to our analysis, this finding seems mainly attributable to the reduction in the time to be placed on the waiting list for PD patients. The tendency to reduce time to transplantation for PD patients was confirmed by the call time to transplant, although the difference in this case was not significant. This finding could be related to a different profile of PD patients. Patients who undergo this method tend to be more empowered, and to pursue their care plan by themselves. Having a strong social support network and being functionally able is strongly associated with choosing PD [27]. This attitude can be translated into a more efficient treatment and a better planning of the examinations necessary for inclusion in the transplantation waiting list. Moreover, these patients have to manage their own treatment daily, which can serve as a strong incentive to obtain quick inclusion in the list. Vice versa, the frequent hospital admissions required for HD may negatively affect the planning of the examinations necessary for inclusion in the transplantation waiting list.

Although not statistically significant, the HD slower time in call-to-transplantation could be partially explained by the fact that these patients had a greater tendency to anemia and more likely required blood transfusion with greater use of erythropoiesis-stimulating agents due to an increase in panel reactive antibody [28]. The presence of antibodies generated by frequent transfusions may therefore hinder the finding of a matching organ for transplantation, prolonging the waiting time in HD patients.

Overall our findings should be taken into account to improve clinical practice and management of modality choice. This may be part of a general improvement strategy aimed to maximize quality of life, patient-reported outcomes, and cost-savings [29, 30].

## Conclusions

The aim of this study was to assess outcomes of survival and time-to-transplantation in the presence of an extensive use of PD in patients with ESRD in the Autonomous Province of Trento. Our data did not show differences in long-term survival between patients treated with PD and with HD. Differently, PD patients seemed to benefit in terms of reduced time for placement on waiting list to transplantation.

Our findings support the wider adoption of PD thanks to its feasibility, and the evidence of positive outcomes and far-reaching benefits for patients. These reported results and the analysis performed should help in devising future studies with similar methods of competing risk survival analysis and management strategies to achieve an improvement in the outcomes of patients undergoing dialysis care.
